# The Association of Smoking Exposure at Home with Attempts to Quit Smoking and Cessation Success: A Survey of South Korean Adolescents Who Smoke

**DOI:** 10.3390/ijerph17114129

**Published:** 2020-06-10

**Authors:** Wonjeong Jeong, Yun Kyung Kim, Jae Hong Joo, Sung-In Jang, Eun-Cheol Park

**Affiliations:** 1 Department of Public Health, Graduate School, Yonsei University, Seoul 03722, Korea; wjjeong@yuhs.ac (W.J.); hykh51128@yuhs.ac (Y.K.K.); jhj3040@yuhs.ac (J.H.J.); 2 Institute of Health Services Research, Yonsei University, Seoul 03722, Korea; jangsi@yuhs.ac; 3 Department of Preventive Medicine, Yonsei University College of Medicine, Seoul 03722, Korea

**Keywords:** smoking exposure at home, South Korean adolescents, attempts to quit smoking, cessation success, KYRBS, smoking adolescents, smoking cessation

## Abstract

This study aimed to examine the association of smoking exposure at home with attempts to quit smoking and the success or failure of such attempts among South Korean adolescents. We utilized the data of 28,652 South Korean adolescents who smoked from the 2015–2017 Korea Youth Risk Behavior Web-based Survey, including demographic variables (age, sex, and family structure), socioeconomic variables (allowance per week, household income level, and grade), and health-related characteristics (alcohol consumption, intensity of physical activity, stress level, self-reported health status, attendance in smoking cessation programs, and smoking onset). A multiple logistic regression analysis revealed that attempting to quit smoking was less likely among those exposed to smoking at home every day compared to those without such exposure (boys exposed to smoking every day: OR = 0.52, CI = 0.45–0.60; girls exposed to smoking every day: OR = 0.48, CI = 0.38–0.61); cessation success showed similar results (boys exposed to smoking every day: OR = 0.51, CI = 0.46–0.58; girls exposed to smoking every day: OR = 0.56, CI = 0.47–0.66). These findings highlight the impacts of smoking exposure at home and the importance of considering this exposure when supporting adolescents to quit.

## 1. Introduction

The World Health Organization cites that smoking is one of the most harmful behaviors that can cause serious health problems [1]. Moreover, tobacco-related death and illness can lead to poverty by forcing people to pay for smoking-related medical expenses [2]. Despite several efforts to prevent adolescent smoking, 9.3% of South Korean boys and 3.8% of girls smoked in 2019 [3]. Adolescent smoking tends to continue into adulthood: 88% of adult daily smokers first used cigarettes at the age of 18 [4,5,6]. Therefore, it is important to reduce lifetime exposure to smoking among adolescents who smoke, in order to make them stop smoking [5,6].

Stopping tobacco use is often considered the most important element in cancer prevention worldwide [7,8,9]. However, smoking cessation programs have had limited success in South Korea, with many smokers unable to abstain from tobacco use for longer than a year [10]. Although many smokers intend to quit, tobacco dependence and the addictive properties of nicotine can render smokers unable to quit [11]. Moreover, a lack of motivation causes many to believe they will never be able to quit smoking [12]. Therefore, the World Health Organization (WHO) announced that tobacco users need help to quit smoking [1]. Counseling and medication can double a tobacco user’s chances of successfully quitting [1,13]. Governments and public health professionals have made intensive efforts to reduce tobacco use, by implementing strong and effective tobacco control policies and measures, such as tobacco tax increases and media campaigns [9,14].

Many of the efforts to reduce tobacco use are aimed at limiting or reducing smoking by young people. Because adolescents are easily influenced by social and environmental factors, family history, and psychosocial and psychopathological problems, many may be motivated to smoke [15,16]. Moreover, for those adolescents who do smoke, peer and familial influences substantially affect their smoking behaviors [16]. However, two-thirds of adolescent smokers worldwide try to quit smoking, but 98% fail within one year [17]. Additionally, living with smokers has been associated with smoking and nicotine addiction among adolescents [18,19], and a previous study showed that high nicotine dependence may negatively affect smoking cessation in this population [20]. As early smoking cessation for adolescents could reduce the prevalence of adults smoking and improve health, it is important to help young people successfully quit smoking [18].

Therefore, it is necessary to investigate the associations between smoking exposure at home and attempts to quit smoking, as well as cessation success, in order to obtain evidence that could help adolescents’ successful smoking cessation. Consequently, the purpose of this study is to examine the association between smoking exposure at home and attempts to quit smoking and the success or failure of such efforts among South Korean adolescents who smoke. We hypothesized that smoking exposure at home is highly associated with decreased attempts to quit smoking and decreased cessation success among adolescents.

## 2. Methods

### 2.1. Data and Study Participants

The 2015–2017 public use files of the Korea Youth Risk Behavior Web-based Survey (KYRBWS) were pooled to obtain a sample of adolescents who reported smoking a cigarette at least once (*n* = 28,652), which was then stratified by sex (boys, *n* = 21,709; girls, *n* = 6943). A total of 194,625 adolescents were included in the KYRBWS (boys, *n* = 99,835; girls, *n* = 94,790). Therefore, 21.7% of boys and 7% of girls were reported smoking a cigarette at least once. The KYRBWS is a cross-sectional survey that has been conducted annually since 2005 by the Korea Centers for Disease Control and Prevention (KCDC), to monitor South Korean health-related behavior among adolescents. The KYRBWS employed an anonymous, Internet-based, self-administered, and structured questionnaire; furthermore, it involved a complex research design. A stratified multistage cluster-sampling design was used to obtain a nationally representative sample of middle and high school adolescents for the survey [21].

The questionnaire consisted of 123 items assessing demographic characteristics and 14 areas of health-related behaviors. The survey’s target population were students in grades 7 through 12 in South Korea. At each grade level, one sample class was chosen, and all students from each of the six sample classes of each school were chosen as the sample students [22]. The KYRBWS follows the ethical standards of the Helsinki Declaration.

The sample was limited only to adolescents who had ever smoked, namely those who had smoked at least once. We excluded those who did not provide information about age, family structure, allowance, household income level, grade, alcohol consumption, intensity of physical activity, stress level, self-reported health status, attending a smoking cessation program at school, or smoking onset. 

### 2.2. Variables

The main dependent variables were attempts to quit smoking, and success of smoking cessation. That is, data regarding ever-smokers (those had smoked at least once) who had made at least one attempt to quit smoking were obtained from responses to the question: “Have you ever attempted to quit smoking?” Attempts to quit smoking were categorized based on whether respondents answered “yes” or “no”. Those who attempted to quit smoking and successfully quit were determined based on responses to the question: “Have you successfully quit smoking for the past 30 days?” The success of smoking cessation was determined based on whether respondents answered “yes” or “no”.

The presence of smoking exposure at home was the main independent variable. That is, self-reported data regarding smoking exposure at home were obtained from responses to the question: “How many days have you been exposed to smoking at your house, in the last seven days?” Individuals who answered “7 days” to the question were placed in the “everyday” group; those who answered “1–6 days” were placed in the “occasionally” group; and “0 days” were placed in the “none” group. Additionally, we included various demographic, socioeconomic, and health-related characteristics. The demographic variables were age (12–15, 16–18), sex (boys, girls), and family structure (both parents, single parent, none). Socioeconomic variables included allowance per week (under 10,000 won, 10,000–20,000 won, 20,000–40,000 won, more than 40,000 won), household income level (low, middle, high), and grade (low, middle, high). Household income level was determined by the self-reported questions about household income level. Grade was determined by the self-reported questions about GPA score. Health-related characteristics included alcohol consumption (ever drink, never), intensity of physical activity (more than 3 days per week, less than 3 days per week), stress level (high, medium, low), self-reported health status (high, medium, low), attending smoking cessation programs (yes, no), and smoking onset (before middle school, during middle school, after middle school). Attendance at smoking cessation programs was determined based on responses to the question: “Have you attended the smoking cessation program at school in the last 12 months?”.

### 2.3. Statistical Analysis

Chi-square tests were conducted to investigate the general characteristics of the study sample. Multiple logistic regression analysis was performed to examine the association of smoking exposure at home, with attempts to quit smoking and cessation success, stratified by sex, after accounting for potential confounders including demographic, socioeconomic, and health-related characteristics. The results were reported as odds ratios (OR), with 95% confidence intervals (CI).

Subgroup analysis was conducted to investigate this association according to stratified self-reported health status, the use of smoking cessation programs at school, and age at smoking onset using a multiple logistic regression analysis. The reason why these three variables were chosen in the subgroup is due to the fact that previous research shows that these mentioned variables are highly associated with attempts to quit smoking and successful smoking cessation [23]. We stratified the participants into subsets based on these three variables, to examine how the relationship between smoke exposure at home and quit attempts/success differs from one characteristic to another. For instance, the probability of quit attempts/success could vary between those who reported high score on self-reported health-status and those who reported low score on self-reported health-status. The subgroup analysis could be useful in determining which characteristics of the participants serve as the effect modifier in explaining the relationship between smoke exposure at home and quit attempts/success. Survey weighting variables were used for all analyses. Differences were considered statistically significant at p-values of <0.05. All analyses were performed using SAS software (version 9.4; SAS Institute Inc., Cary, NC, USA).

## 3. Results

Table 1 presents the general characteristics of the study sample. Among the participants (21,709 boys and 6943 girls), 18,869 boys (86.9%) and 6206 girls (89.4%) had attempted to quit smoking. Regarding smoking cessation, 11,887 boys (54.8%) and 4329 girls (62.4%) had successfully stopped smoking. In addition, the frequency of attempts to quit smoking and cessation success generally varied according to demographic, socioeconomic, and health-related characteristics.

Table 2 shows the association of smoking exposure at home, with attempts to quit smoking and cessation success. Compared to boys and girls who were never exposed to smoking, those who were exposed to smoking at home every day, or occasionally, had lower odds of attempting to quit smoking (boys exposed to smoking every day: OR = 0.51, CI = 0.44–0.59; girls exposed to smoking every day: OR = 0.48, CI = 0.38–0.61), and had lower cessation success rates (boys exposed to smoking every day: OR = 0.51, CI = 0.45–0.57; girls exposed to smoking every day: OR = 0.56, CI = 0.47–0.67). Adolescents who never drank alcohol had higher odds of attempting to quit smoking and of cessation success than those who drank alcohol. Adolescents who attended the smoking cessation program at school had higher odds of attempting to quit smoking than those who did not attend (boys who attended the smoking cessation program: OR = 1.81, CI = 1.66–1.97; girls who attended the smoking cessation program: OR = 1.57, CI = 1.31–1.88). 

Table 3 shows the results of the subgroup analysis, representing the odds ratio of attempts to quit smoking, stratified by smoking exposure at home. For all levels of self-reported health status, boys who were exposed to smoking at home every day had lower odds of attempting to quit smoking than those who were never exposed to smoking at home (boys exposed to smoking every day: high self-reported health status, OR = 0.55, CI = 0.47–0.65; moderate self-reported health status, OR = 0.46, CI = 0.35–0.62; low self-reported health status, OR = 0.44, CI = 0.27–0.71). For both those who attended the smoking cessation program at school and those who did not, adolescents exposed to smoking at home every day had lower odds of attempting smoking cessation than those who were never exposed to smoking at home. Moreover, there were lower odds of attempts to quit smoking among all levels of smoking onset and who were exposed to smoking everyday than among those who were never exposed to smoking at home. 

Table 4 shows the results of the subgroup analysis, representing the odds ratio of smoking cessation, stratified by smoking exposure at home. For all levels of self-reported health status, boys who were exposed to smoking at home every day had lower odds of smoking cessation than those who were never exposed to smoking at home (boys exposed to smoking every day: high self-reported health status, OR = 0.52, CI = 0.46–0.60; moderate self-reported health status, OR = 0.46, CI = 0.36–0.60; low self-reported health status, OR = 0.52, CI = 0.34–0.80). For both those who attended the smoking cessation program at school and those who did not, adolescents exposed to smoking at home every day had lower odds of successful smoking cessation than those who were never exposed to smoking at home. Moreover, for all levels of smoking onset, boys who are exposed to smoking everyday had lower odds of smoking cessation than those who were never exposed to smoking at home. 

## 4. Discussion

The main aim of this study was to clarify the association of smoking exposure at home with attempts to quit smoking and cessation success among South Korean adolescents who smoke. Our findings show that, compared to boys and girls who were never exposed to smoking, those who were exposed to smoking at home every day or occasionally had lower odds of attempting to quit smoking and lower odds of cessation success. 

Smoking exposure at home creates psychosocial exposure to family smoking and physiological exposure to nicotine for adolescents [18,24,25], and adolescent smoking cessation is highly associated with parenting role models [24,25]. Previous studies have shown that seeing family members smoke could constitute a smoking cue, which can increase nicotine craving [18,25]. Additionally, covert smoking of the adolescent at home may be more difficult to detect amid the smoking of other family members, which could make it easy for them to “get away with” smoking [18]. Moreover, as smoking cessation in adolescents is highly associated with parental disapproval of their smoking, smoking exposure at home could lower their attempt to quit smoking [24,25]. Previous studies have also shown that whether or not an adolescent smokes is more highly impacted by the context of smoking exposure at home than by school influences such as peer smoking [26,27]. Moreover, not only exposure smoking from parents, but also smoking exposure from older siblings, can be highly impactful on adolescents [28]. Allowing smoking in the home may create an environment where siblings or peers who smoke are more likely to interact [28,29]. Moreover, when they are exposed to smoking at home, adolescents are more likely to become daily smokers, and more effort is required for a daily smoker to quit smoking than for an occasional smoker to quit [28]. It is therefore not unexpected that smoking exposure at home is highly associated with both being unlikely to attempt cessation and being unlikely to succeed at quitting for those who try.

Smoking cessation among South Korean adolescents has been examined previously [23,30,31], and study results have indicated that adolescents who smoke usually start smoking when they experience considerable academic stress and have trouble in their relationships with parents or friends [30,31]. Prior research has also shown that higher self-efficacy scores relate to higher probabilities of success in quitting smoking, and that the earlier someone starts smoking, the harder it is to successfully quit [23]. The more a child is exposed to parents’ smoking at home, or the more they experience high academic stress, the harder it is for them to succeed in quitting smoking [23,30,31]. Additionally, when adolescents are exposed to smoking at home, they are more likely to become “regular” rather than “experimental” smokers, and the former need considerably more time and effort to quit smoking [27]. In the current study, we also found that the earlier young people start smoking and are exposed to smoking at home every day, the lower their odds of successful smoking cessation. We also found that starting smoking in high school, having higher academic stress, and being exposed to smoking at home are all associated with lower odds of successful smoking cessation.

Adolescents are also more likely to struggle with successfully quitting smoking, because they have less self-control than adults; however, the help of parents and teachers can greatly increase their chances of successful smoking cessation [31,32,33]. Likewise, smoking cessation programs can improve rates of quitting smoking [23,30]. In the current study, we found that attending smoking cessation programs was associated with higher odds of attempting to quit smoking; however, we also found lower odds of successful smoking cessation associated with attending smoking cessation programs [32,33]. The lack of success from the programs may be due to the fact, as previous studies have shown, that smoking cessation programs in schools for Korean adolescents are mostly conducted only once a year and are inconsistent because there are no proper program guidelines [32]. In addition, the programs were found to lack any means of directly helping adolescents with smoking, because they relied on simple knowledge transfer and attitude changes [33]. The lack of successful cessation programs for adolescents is especially important, because research has also shown that those who have attempted and failed to quit are likely to smoke even more frequently (more per day or per week) than those who have not tried to quit [34]. This result demonstrates that more delicate care is needed for those who fail to quit smoking [34]. Therefore, it is necessary to ensure that those who attempt to quit smoking can succeed, which requires care, and a continuous smoking cessation program can be helpful [32]. The current smoking cessation programs may be helpful for some adolescents trying to quit smoking, but proper established guidelines for a continuous and effective smoking cessation program in schools are needed for greater success [32,33].

Although this study produced some compelling results, it also has several limitations. First, the results were derived from self-reported data; thus, the data from girls who smoke is liable to be less than entirely correct. As shown by a previous study in Korea, females tend to hide their smoking [35]. Therefore, some girls who smoke may have answered that they do not smoke, which implies that there could be more girls who smoke. Therefore, for the data regarding girls and smoking, comparisons with boys’ data might be better for interpreting the results. Second, we did not have any information regarding individuals’ numbers of attempts to cease smoking, because this information was not contained in the data set from the original study. Thus, no distinction was made between those who had previously tried to quit smoking and those who were trying to quit for the first time. Therefore, we could not assess the relationship between the number of previous attempts to quit smoking and cessation success. Finally, our study lacked information about those who successfully quit smoking. We could not determine how long they smoked before they quit smoking, because the data set did not contain that information. We realize that the lack of information on the number of quit attempts and the identity of those who quit smoking is an important limitation of the study, and thus, further investigation with a more detailed survey is suggested, to validate the findings of our study. 

Despite these limitations, our study has numerous strengths. The KYRBWS is representative of the overall adolescent student population in grades 7 through 12. The survey used an elaborate design that included multistage sampling, stratification, and clustering. Therefore, the results can be generalized to the overall adolescent population in South Korea. Furthermore, as the KYRBWS was a web-based anonymous survey, obtaining honest responses was relatively likely. Moreover, the results from the current study can be used as a baseline for increasing the adolescent smoking cessation rate. 

For adolescents who smoke to stop successfully, they need help from people around them. First, schools should continue to educate smoking adolescents, through better quality smoking cessation programs with constant monitoring. It is important to pay attention to the home environments of adolescents who smoke, to support them in quitting smoking and not being exposed to smoking. Cessation plans could be better if parents and their adolescents quit together, as young people are heavily affected by their parents’ smoking [18,24,25]. Moreover, government and public health professionals should engage in intensive efforts to reduce tobacco use, by developing tobacco control policies (e.g., legislation to ban smoking), media campaigns, educational exhibitions, and public service announcements [9,14,36,37]. Above all, it is important for adolescents to realize the need to quit smoking and be alert to this need. 

## 5. Conclusions

The current study identified a significant association between smoking exposure at home, with lower attempts to quit smoking and lower successful smoking cessation rates. Our finding suggests that adolescents who are exposed to smoking at home, every day or occasionally, are less likely to attempt to quit smoking and less likely to achieve smoking cessation success. As adolescent smoking can lead to premature death, it is important to support adolescents’ successful smoking cessation [16]. These results, taken together, highlight the association of smoking exposure at home, with lower attempts to quit smoking and lower success rates for those who attempt cessation. Thus, the current study may be used to motivate parents and/or other family members to not smoke near adolescents. Moreover, this study may motivate adolescents who smoke to quit successfully by attending an improved quality smoking cessation program. 

## Figures and Tables

**Table 1:** General characteristics of the study population.

Variables	Boys (N = 21,709)					Girls (N = 6943)				
	Total	Attempts to Quit Smoking		Smoking Cessation		Total	Attempts to Quit Smoking		Smoking Cessation	
		N	(%)	N	(%)		N	(%)	N	(%)
**Smoking Exposure at Home**										
Everyday	1928	1512	(78.4)	725	(37.6)	946	794	(83.9)	484	(51.2)
Occasionally	6029	5236	(86.8)	3110	(51.6)	2202	1956	(88.8)	1276	(57.9)
None	13,752	12,121	(88.1)	8052	(58.6)	3795	3456	(91.1)	2569	(67.7)
**Age**										
12–15	8246	7465	(90.5)	5291	(64.2)	2701	2437	(90.2)	1759	(65.1)
16–18	13,463	11,404	(84.7)	6596	−49	4242	3769	(88.8)	2570	(60.6)
**Having Parents**										
Both parents	19,864	17,365	(87.4)	11,046	(55.6)	6194	5568	(89.9)	3946	(63.7)
Single parent	1436	1202	(83.7)	693	(48.3)	602	521	(86.5)	341	(56.6)
None	409	302	(73.8)	148	(36.2)	147	117	(79.6)	42	(28.6)
**Allowance**										
Low	3686	3296	(89.4)	2399	(65.1)	1198	1086	(90.7)	853	(71.2)
Lower-middle	5253	4705	(89.6)	3282	(62.5)	1552	1418	(91.4)	1089	(70.2)
Upper-middle	5927	5167	(87.2)	3160	(53.3)	1878	1669	(88.9)	1135	(60.4)
High	6843	5701	(83.3)	3046	(44.5)	2315	2033	(87.8)	1252	(54.1)
**Household Income Level**										
Low	4546	3936	(86.6)	2324	(51.1)	1942	1722	(88.7)	1167	(60.1)
Middle	9659	8468	(87.7)	5534	(57.3)	3133	2818	(89.9)	2010	(64.2)
High	7504	6465	(86.2)	4029	(53.7)	1868	1666	(89.2)	1152	(61.7)
**Grade**										
High	6132	5364	(87.5)	3777	(61.6)	1684	1491	(88.5)	1138	(67.6)
Middle	5595	4938	(88.3)	3194	(57.1)	1627	1490	(91.6)	1120	(68.8)
Low	9982	8567	(85.8)	4916	(49.2)	3632	3225	(88.8)	2071	−57
**Alcohol Consumption**										
Never	3949	3627	(91.8)	2990	(75.7)	865	818	(94.6)	716	(82.8)
Ever	17,760	15,242	(85.8)	8897	(50.1)	6078	5388	(88.6)	3613	(59.4)
**Physical Activity**										
Low	10,287	8797	(85.5)	5492	(53.4)	5107	4534	(88.8)	3183	(62.3)
High	11,422	10,072	(88.2)	6395	−56	1836	1672	(91.1)	1146	(62.4)
**Stress Level**										
High	7747	6614	(85.4)	3924	(50.7)	4069	3608	(88.7)	2428	(59.7)
Medium	9464	8344	(88.2)	5391	−57	2230	2009	(90.1)	1478	(66.3)
Low	4498	3911	(86.9)	2572	(57.2)	644	589	(91.5)	423	(65.7)
**Self-Reported Health Status**										
High	16,330	14,311	(87.6)	9135	(55.9)	3910	3513	(89.8)	2515	(64.3)
Medium	4120	3501	−85	2139	(51.9)	2146	1900	(88.5)	1298	(60.5)
Low	1259	1057	−84	613	(48.7)	887	793	(89.4)	516	(58.2)
**Smoking Cessation Program at School**										
Yes	13,980	12,531	(89.6)	7638	(54.6)	4432	4031	−91	2692	(60.7)
No	7729	6338	−82	4249	−55	2511	2175	(86.6)	1637	(65.2)
**Smoking Onset**										
Elementary school	5943	5132	(86.4)	3289	(55.3)	1778	1605	(90.3)	1142	(64.2)
Middle school	13,559	11,908	(87.8)	7451	−55	4222	3799	−90	2632	(62.3)
High school	2207	1829	(82.9)	1147	−52	943	802	−85	555	(58.9)
**Year**										
2015	8608	7484	(86.9)	4612	(53.6)	2801	2540	(90.7)	1849	−66
2016	7142	6249	(87.5)	4070	−57	2182	1939	(88.9)	1373	(62.9)
2017	5959	5136	(86.2)	3205	(53.8)	1960	1727	(88.1)	1107	(56.5)
**TOTAL**	**21,709**	**18,869**	**(86.9)**	**11,887**	**(54.8)**	**6943**	**6206**	**(89.4)**	**4329**	**(62.4)**

**Table 2:** Factors associated with attempts to quit smoking and smoking cessation.

Variables	Boys				Girls			
	Attempts to Quit Smoking		Smoking Cessation		Attempts to Quit Smoking		Smoking Cessation	
	Adjusted OR	95% CI	Adjusted OR	95% CI	Adjusted OR	95% CI	Adjusted OR	95% CI
**Smoking Exposure at Home**								
Everyday	0.51	(0.44–0.59)	0.51	(0.45–0.57)	0.48	(0.38–0.61)	0.56	(0.47–0.67)
Occasionally	0.87	(0.79–0.97)	0.78	(0.73–0.84)	0.74	(0.61–0.90)	0.71	(0.62–0.80)
None	1.00		1.00		1.00		1.00	
**Age**								
12–15	1.46	(1.31–1.63)	1.53	(1.42–1.65)	0.93	(0.76–1.13)	1.02	(0.89–1.17)
16–18	1.00		1.00		1.00		1.00	
**Having Parents**								
Both parents	2.27	(1.77–2.92)	2.21	(1.71–2.87)	2.3	(1.46–3.61)	4.9	(3.26–7.36)
Single parent	1.75	(1.31–2.33)	1.73	(1.30–2.30)	1.85	(1.11–3.08)	4.08	(2.62–6.34)
None	1.00		1.00		1.00		1.00	
**Allowance**								
Low	1.35	(1.18–1.55)	1.92	(1.74–2.12)	1.2	(0.93–1.57)	1.87	(1.58–2.22)
Lower-middle	1.39	(1.22–1.57)	1.73	(1.59–1.89)	1.37	(1.08–1.75)	1.74	(1.48–2.03)
Upper-middle	1.2	(1.07–1.34)	1.3	(1.20–1.41)	1	(0.81–1.23)	1.23	(1.07–1.41)
High	1.00		1.00		1.00		1.00	
**Household Income Level**								
Low	1.2	(1.06–1.36)	1.08	(0.98–1.19)	0.93	(0.73–1.18)	1.07	(0.91–1.27)
Middle	1.14	(1.02–1.26)	1.21	(1.13–1.30)	1.03	(0.84–1.27)	1.13	(0.98–1.30)
High	1.00		1.00		1.00		1.00	
**Grade**								
High	1.11	(0.99–1.24)	1.67	(1.54–1.80)	0.88	(0.72–1.07)	1.6	(1.39–1.85)
Middle	1.21	(1.08–1.36)	1.35	(1.25–1.46)	1.17	(0.94–1.47)	1.54	(1.33–1.79)
Low	1.00		1.00		1.00		1.00	
**Alcohol Consumption**								
Never	1.59	(1.38–1.83)	2.61	(2.38–2.86)	2	(1.43–2.80)	3.07	(2.47–3.80)
Ever	1.00		1.00		1.00		1.00	
**Physical Activity**								
Low	0.86	(0.79–0.94)	0.99	(0.93–1.06)	0.84	(0.69–1.03)	1.07	(0.94–1.22)
High	1.00		1.00		1.00		1.00	
**Stress Level**								
High	1	(0.88–1.14)	0.84	(0.78–0.92)	0.75	(0.53–1.06)	0.81	(0.66–1.00)
Medium	1.17	(1.03–1.31)	1.02	(0.94–1.11)	0.79	(0.55–1.14)	1.04	(0.83–1.30)
Low	1.00		1.00		1.00		1.00	
**Self-Reported Health Status**								
High	1.14	(0.96–1.36)	1.08	(0.94–1.24)	0.86	(0.65–1.13)	1.09	(0.91–1.31)
Medium	1.02	(0.85–1.24)	1.02	(0.88–1.18)	0.85	(0.64–1.14)	0.99	(0.81–1.20)
Low	1.00		1.00		1.00		1.00	
**Smoking Cessation Program at School**								
Yes	1.81	(1.66–1.97)	0.93	(0.88–1.00)	1.57	(1.31–1.88)	0.82	(0.73–0.93)
No	1.00		1.00		1.00		1.00	
**Smoking Onset**								
Elementary school	1.19	(1.01–1.39)	0.95	(0.85–1.07)	1.67	(1.27–2.21)	1.08	(0.88–1.32)
Middle school	1.35	(1.17–1.55)	0.97	(0.87–1.08)	1.51	(1.19–1.90)	1.03	(0.87–1.21)
High school	1.00		1.00		1.00		1.00	
**Year**								
2015	1.09	(0.97–1.22)	0.91	(0.83–1.01)	1.27	(1.02–1.57)	1.32	(1.12–1.54)
2016	1.12	(0.99–1.26)	1.08	(0.97–1.19)	1.09	(0.86–1.38)	1.28	(1.09–1.50)
2017	1.00		1.00		1.00		1.00	

**Table 3:** Subgroup analysis representing the odds ratios for attempts to quit smoking, stratified by smoking exposure at home.

Variables	Exposed to Smoking Everyday		Exposed to Smoking Occasionally		Never Exposed
	Adjusted OR	95% CI	Adjusted OR	95% CI	Adjusted OR
**Boys**					
**Self-Reported Health Status**					
High	0.55	(0.47–0.65)	0.86	(0.77–0.96)	1.00
Medium	0.46	(0.35–0.62)	0.91	(0.72–1.15)	1.00
Low	0.44	(0.27–0.71)	0.94	(0.63–1.41)	1.00
**Smoking Cessation Program at School**					
Yes	0.54	(0.45–0.66)	0.83	(0.73–0.95)	1.00
No	0.48	(0.39–0.59)	0.93	(0.80–1.09)	1.00
**Smoking Onset**					
Elementary school	0.49	(0.38–0.62)	0.88	(0.73–1.05)	1.00
Middle school	0.52	(0.43–0.63)	0.9	(0.78–1.03)	1.00
High school	0.59	(0.38–0.90)	0.81	(0.61–1.09)	1.00
**Girls**					
**Self-Reported Health Status**					
High	0.47	(0.33–0.65)	0.71	(0.55–0.92)	1.00
Medium	0.57	(0.37–0.87)	0.79	(0.57–1.11)	1.00
Low	0.41	(0.23–0.73)	0.75	(0.42–1.34)	1.00
**Smoking Cessation Program at School**					
Yes	0.51	(0.37–0.69)	0.7	(0.54–0.91)	1.00
No	0.45	(0.31–0.64)	0.78	(0.58–1.07)	1.00
**Smoking Onset**					
Elementary school	0.45	(0.29–0.70)	0.91	(0.60–1.39)	1.00
Middle school	0.5	(0.37–0.69)	0.82	(0.63–1.06)	1.00
High school	0.51	(0.27–0.98)	0.46	(0.31–0.69)	1.00

**Table 4:** Subgroup analysis, representing the odds ratios for smoking cessation, stratified by smoking exposure at home.

Variables	Exposed to Smoking Everyday		Exposed to Smoking Occasionally		Never Exposed
	Adjusted OR	95% CI	Adjusted OR	95% CI	Adjusted OR
**Boys**					
**Self-Reported Health Status**					
High	0.52	(0.46–0.60)	0.79	(0.73–0.86)	1.00
Medium	0.46	(0.36–0.60)	0.7	(0.59–0.83)	1.00
Low	0.52	(0.34–0.80)	1.05	(0.78–1.42)	1.00
**Smoking Cessation Program at School**					
Yes	0.51	(0.44–0.59)	0.82	(0.75–0.90)	1.00
No	0.5	(0.42–0.60)	0.72	(0.64–0.81)	1.00
**Smoking Onset**					
Elementary school	0.43	(0.35–0.53)	0.68	(0.59–0.78)	1.00
Middle school	0.56	(0.48–0.64)	0.84	(0.76–0.92)	1.00
High school	0.45	(0.32–0.65)	0.8	(0.64–1.01)	1.00
**Girls**					
**Self-Reported Health Status**					
High	0.5	(0.39–0.64)	0.63	(0.53–0.74)	1.00
Medium	0.73	(0.54–0.98)	0.84	(0.67–1.04)	1.00
Low	0.52	(0.34–0.79)	0.79	(0.56–1.12)	1.00
**Smoking Cessation Program at School**					
Yes	0.52	(0.43–0.64)	0.74	(0.63–0.86)	1.00
No	0.64	(0.48–0.84)	0.65	(0.52–0.82)	1.00
**Smoking Onset**					
Elementary school	0.57	(0.41–0.80)	0.48	(0.37–0.63)	1.00
Middle school	0.6	(0.48–0.74)	0.87	(0.74–1.02)	1.00
High school	0.36	(0.22–0.58)	0.58	(0.42–0.81)	1.00
